# Differentiation roadmap of embryonic Sertoli cells derived from mouse embryonic stem cells

**DOI:** 10.1186/s13287-019-1180-6

**Published:** 2019-03-08

**Authors:** Chenze Xu, Ali Mohsin, Yanxia Luo, Lili Xie, Yan Peng, Qizheng Wang, Haifeng Hang, Yingping Zhuang, Meijin Guo

**Affiliations:** 1 State Key Laboratory of Bioreactor Engineering, East China University of Science and Technology, 130 Meilong Rd., Shanghai, 200237 People’s Republic of China; 2 Engineering Research Centre of Processes System, Ministry of Education, East China University of Science and Technology, 130 Meilong Rd., Shanghai, 200237 People’s Republic of China

**Keywords:** Embryonic stem cells, Embryonic Sertoli cells, Lentiviral transduction, Molecular mechanism, Gonadogenesis, Male determinant factors

## Abstract

**Background:**

Embryonic Sertoli cells (eSCs) play an important role in sex determination and in male gonad development which makes them a very useful cell type for therapeutic applications. However, the deriving mechanism of Sertoli cells has been unclear and challenging to create a large number of quality eSCs. Therefore, this study aimed to create the eSCs induced from mouse embryonic stem (mES) cells by regulating defined factors and to explore the relevant regulatory mechanism.

**Methods:**

Six inducing factors, Sry, Sox9, SF1, WT1, GATA4, and Dmrt1, were respectively transduced into mES cells by lentiviral infection according to the experimental design. The test groups were identified by development stage-specific markers, AMH, Emx2, SF1, and FasL, using flow cytometry. Induced eSCs were determined by FasL and AMH biomarkers under immunofluorescence, immunocytochemistry, and flow cytometry. Moreover, the pluripotency markers, gonad development-related markers, epithelial markers and mesenchymal markers in test groups were transcriptionally determined by qPCR.

**Results:**

In this study, the co-overexpression of all the six factors effectively produced a large population of eSCs from mES cells in 35 days of culturing. These eSCs were capable of forming tubular-like and ring-like structures with functional performance. The results of flow cytometry indicated that the upregulation of GATA4 and WT1 contributed to the growth of somatic cells in the coelomic epithelium regarded as the main progenitor cells of eSCs. Whereas, SF1 facilitated the development of eSC precursor cells, and Sry and Sox9 promoted the determination of male development. Moreover, the overexpression of Dmrt1 was essential for the maintenance of eSCs and some of their specific surface biomarkers such as FasL. The cellular morphology, biomarker identification, and transcriptomic analysis aided in exploring the regulatory mechanism of deriving eSCs from mES cells.

**Conclusion:**

Conclusively, we have elucidated a differentiation roadmap of eSCs derived from mES cells with a relevant regulatory mechanism. Through co-overexpression of all these six factors, a large population of eSCs was successfully induced occupying 24% of the whole cell population (1 × 10^5^ cells/cm^2^). By adopting this approach, a mass of embryonic Sertoli cells can be generated for the purpose of co-culture technique, organ transplantation, gonadal developmental and sex determination researches.

**Electronic supplementary material:**

The online version of this article (10.1186/s13287-019-1180-6) contains supplementary material, which is available to authorized users.

## Introduction

Sertoli cells, the first male-specific cells generated during embryo development which play an important role in male determination and in male gonadal development, have been widely studied regarding their functions in immunosuppression, male reproductive development, and co-culture technique [1–3]. However, the deriving mechanisms of Sertoli cells remain unresolved [4–9].

Sertoli cells are of great value in co-culture technique to provide immunosuppression for tissue transplant [10]; to improve the survival and proliferation of neurons [11, 12], mesenchymal stem cells [3, 13], islets [1], endothelial cells [14], and hepatocytes [15]; and to promote the maturation of spermatogonial stem cells (SSCs) [16, 17]. However, it is difficult to produce a large number of Sertoli cells by using conventional methods. Mature Sertoli cells, being mitosis inactive, and TM4 cells, a semi-permanent engineered Sertoli cell line, are not suitable for developmental mechanism studies, clinical applications, and co-culture techniques [7, 18]. It has been demonstrated that eSCs are well functional in supporting other cells and superior in supporting the development of SSCs as feeder cells compared to mature Sertoli cells or TM4 cells [17]. Therefore, eSCs have great potential in basic research studies and clinical applications. Unfortunately, it is difficult to obtain a large number of eSCs from embryos [6, 17]. To generate eSCs, some studies improved the population of Sertoli cells from human embryonic stem cells by reducing the size of the cultured cell colonies [19], or generated embryonic Sertoli-like cells transdifferentiated from mouse fibroblasts [20]. However, the regulation of cell colonies referred to repeated FACS, which might results in cell loss and damage. Moreover, the induced eSCs transdiffered from fibroblast have some limitations to stimulate the in vivo generation of Sertoli cells.

In this scenario, we hold the opinion that the novel efficient eSC-inducing approaches established on a mouse embryonic stem (mES) cell developmental model can contribute to future research studies, such as in co-culture or co-transplant techniques. Regarding the in vivo developmental process leading to Sertoli cells, the most widely accepted theory indicates that the SF1-positive somatic cells in coelomic epithelial are the main precursor cells of pre-Sertoli cells [21]. Firstly, the somatic cells in coelomic epithelial originate from the mesoderm involving the expression of SF1 [22], WT1 (−KTS isoform) [9, 23], GATA4 [9, 24, 25], Lhx9 [26], Emx2 [27], Pod1 [9], TIF1β [9], TIF2 [9], and Insr [9]. Secondly, these somatic cells migrate into bi-potential gonads referring to the expression of SIX1/4 [28], FOG2/GATA4 [24], Cbx2 [29], Map3k4 [30, 31], and Gadd45g [32]. Thirdly, a part of these somatic cells develop into SF1-positive precursor cells [21], and then pre-Sertoli cells undergo the influence of SF1 [22], WT1 (−KTS isoform) [23], Sry [5], Sox9 [24, 33], and Sox8 [34]. Finally, to maintain male development, the presence of several factors like FGF9/FGFR2 [35, 36], PGD2 [37]**,** AMH [38], and Dmrt1 [39, 40] is necessary. Additionally, some signals promoting female development, such as RSPO1 [41], WNT4 [42], DAX1 [43], FOXL2 [44], and β-catenin [41], must be inhibited.

Based on the previous research literature, we screened and selected six potential inducing factors, Sry, Sox9, SF1, WT1, GATA4, and Dmrt1, intending to establish a reductionist approach to reprogram the developmental process from mES cells to eSCs [6, 8, 9, 23–25, 34, 39, 45–48]. Firstly, we managed to improve the culturing approaches of mES cells by replacing the mouse embryo fibroblast (MEF) feeder with TM4 feeder cells, providing a big number of quality mES cells. With cotransduction of all the six factors, eSCs were successfully induced from these mES cells. After that, the factors were co-upregulated in mES cells with different transcription factors and were removed in each test group to determine their individual function; this approach provided significant evidence through indicating the discrete roles played by these inducing factors and further relieved the developmental barriers for generating eSCs. The maximum cell portion of eSCs (AMH^+^/FasL^+^) reached 23.7% in the transduced groups (group All factors, i.e., mES+Trans) (1 × 10^5^ cells/cm^2^) and up to 56.1% with the pebble-like colonies (PCs) removed by mechanical isolation. These generated eSCs were capable of forming tubular-like structures at high cell densities and ring-like structures at low cell densities. Finally, we detected the cell population, morphological changes, transcriptional level of major biomarkers, timing of epithelial-mesenchymal transformation (EMT) and mesenchymal-epithelial transformation (MET), and finally determined the developmental process from mES cells to eSCs. Conclusively, we proposed a roadmap involving key factors, developmental stages, and specific biomarkers, which established the foundation of revealing the mechanism of inducing eSCs from mES cells.

## Materials and methods

### Preparation of lentivirus

To obtain the six target genes, Sry, Sox9, SF1, WT1, GATA4, and Dmrt1, they were selectively amplified by PCR from the mouse whole DNA sequences (extracted by a TIANamp Genomic DNA Kit (TIANGEN, China)) or totally synthesized by Takara (Japan) (genes like Sry have multiple repetitive sequences were hard to amplified by PCR). Primer design is listed in Additional file 1: Table S2. To produce lentiviral plasmids, the six inducing factors were separately integrated into FUW-TetO vectors between the restriction enzyme sites *Bam*H1 and *Eco*R1 and were constructed into six plasmids (FUW-TetO-Sox9, FUW-TetO-WT1, FUW-TetO-GATA4, FUW-TetO-Sry, FUW-TetO-SF1, and FUW-TetO-Dmrt1). The constructed plasmids were amplified in DH5α *E. coli* and later extracted by an EndoFree Mini Plasmid Kit II (TIANGEN, China).

HEK293T cells were cultured in Opti-MEM (Gibco, USA). Following the manufacturer’s instructions, each group of HEK293T cells was separately transfected with one of the six plasmids (FUW-TetO-Sox9, FUW-TetO-WT1, FUW-TetO-GATA4, FUW-TetO-Sry, FUW-TetO-SF1, or FUW-TetO-Dmrt1) and respectively co-transfected with psPAX2 and PMD.2G by Lipofectamine3000 (Thermo, USA) (Additional file 1: Table S4). The supernatant was collected after 48–72 h of post-transfection and was concentrated with Lenti-Pac™ Lentivirus Concentration Solution (GeneCopoeia, USA), followed by its storage − 80 °C for later use.

### mES cell line and culture

The mouse mES cells used in the current study were derived from R1/E cell line (male gender, 129X1 × 129S1), and mouse embryo fibroblasts (MEFs) were derived from Kunming white mice between 12.5 and 13.5 dpc. Both cell lines were obtained from the Chinese Academy of Sciences cell bank (Shanghai, China).

To culture mES cells, MEFs (passage 3, P3) treated with mitomycin C (10 μg/ml, 2–3 h) were seeded in 0.1% gelatin-coated T-flasks as feeder layers. TM4 cells cultured with mES cells as feeder were treated with mitomycin C according to their confluence (Additional file 1: Table S1). After 12–24 h, mES cells were recovered from nitrogen cryopreservation using medium composed of DMEM with 12.5% fetal calf serum (FBS), 0.11 g/L sodium pyruvate, 0.30 g/L L-glutamine, 1.5 g/L sodium bicarbonate, 0.5 g/L HEPES, 50.0 μmol β-mercaptoethanol, 1× non-essential amino acids (NEAA), and 10^3^ U/mL leukemia inhibitory factor (LIF). Culture medium was replaced every day.

In differentiation experiments, LIF and β-mercaptoethanol were removed from the culture medium as the inducing medium at day 5. Inducing medium was replaced every 2 days. Cell passages were performed when cell confluence reaches over 80%, and cell dissociation was conducted using collagenase I (Gibco, USA).

### qPCR (quantitative RT-PCR)

Total RNA from the test groups was isolated using Invitrogen™ TRIzol™ (Thermo, USA), then reverse-transcribed by a PrimeScript™ RT reagent Kit with gDNA Eraser (Perfect Real Time) (TAKARA, Japan). qPCR was performed with SYBR Premix Ex Taq™ II (Tli RNaseH Plus) (TAKARA, Japan) according to the manufacturer’s instructions on a CFX96 touch qPCR system (Bio-Rad, USA). Primer design is listed in Additional file 1: Table S3.

### Immunofluorescence (IF) and immunocytochemistry (ICC)

The cell samples being fixed with 4.0% methanol (10-30 min) were perforated on the membrane by Triton X-100 (0.1%, for less than 10 min) and were washed with PBS for three times (10 min per wash). Later, they were blocked with 5% bovine serum albumin (BSA) for 30 min and were incubated with antibodies and Dapi (Sigma, USA) according to the manufacturer’s instruction. Followed by washing with PBS as above, the samples were incubated with secondary antibodies, before being completely ready for observation under an EVOS FL Auto imaging system (Life Technologies, USA). The antibodies used in this work are listed in Additional file 1: Table S5.

### Flow cytometry (FCM) analysis

Cell samples were dissociated by 0.25% trypsin-EDTA and were washed with PBS followed by their perforation on the membrane by Triton X-100 (0.1%, for less than 10 min), and later were washed again with PBS and were quantified. Then samples were re-suspended in a 100-μL volume of DMEM in a concentration of 1 × 10^6^–10^7^ cells/mL. Matched controls of antibody for FCM were applied according to the manufacturer’s instructions using a FACSArial system (BD Biosciences, USA). The quad was set according to isolated mature Sertoli cells, group No factor (control group, i.e., mES+MEF), and group All factors (mES+Trans) according to FasL, AMH, and Sox9 (Additional file 1: Figure S2A, B, C, D, E). Antibodies are listed in Additional file 1: Table S6.

### Statistical analysis

For experiments replacing MEF with TM4 cells, test samples were cultured in T25 flasks with three parallel samples in each group. In qPCR, results are the average mean of three to four tests for each sample. In experiments inducing eSCs from mES cells, the experiments were successively repeated three to four times. In each experiment, every test group was cultured in T25 flasks with three to four parallel samples. Error bars represent ±SD (standard deviation). Reliable data meet the condition SD/mean < 10%. Experimental data are reported as mean ± SD. Heat map was expressed as mean value (*n* = 3).

Asterisks indicate statistical significance which was evaluated by one-way ANOVA with SPSS software, and *P* values < 0.05 were considered statistically significant (*), *P* values < 0.01 have great significant statistical difference (**), and *P* values < 0.001 have extreme great significant statistical difference (***).

## Results

### Mitomycin-treated TM4 cells can replace MEF as feeder cells of mES cells to improve the cells’ pluripotency and gonadal differentiation potential

Generally, mES cells require MEFs as feeder cells to recover from nitrogen cryopreservation and to stimulate the mES growth in vitro. However, mES cells cultured on MEF feeder layers usually have relatively poor growth rates, low cell resuscitation rates after liquid nitrogen cryopreservation, and lose pluripotency over time [49, 50]. Moreover, MEF feeder layers are mainly isolated from mice embryos, as being unstable in different donor sources. Thus, all these kinds of limitations hamper their application for the large-scale production of mES cells using MEFs as feeder layers. In order to obtain large numbers of quality mES cells for later use, we sought alternative culture methods to improve their proliferation and pluripotency. TM4 cells, a genetically engineered Sertoli cell line, are capable of being cultured for dozens of passages, secreting trophic proteins to facilitate the survival, proliferation, and differentiation of other cells [3, 43], and could be considered to provide a beneficial environment for mES cells to differentiate into eSCs. Therefore, we used TM4 cells as the feeder layers for culturing of mES cells.

To produce TM4 feeder cells, TM4 cells were treated with mitomycin C according to the conditions presented in Additional file 1: Table S1. Most of the treated TM4 cells encountered apoptosis within 2 weeks. Moreover, no obvious proliferation of TM4 cells was observed in 1 month when cultured alone or when co-cultured with mES cells.

**Fig. 1:**
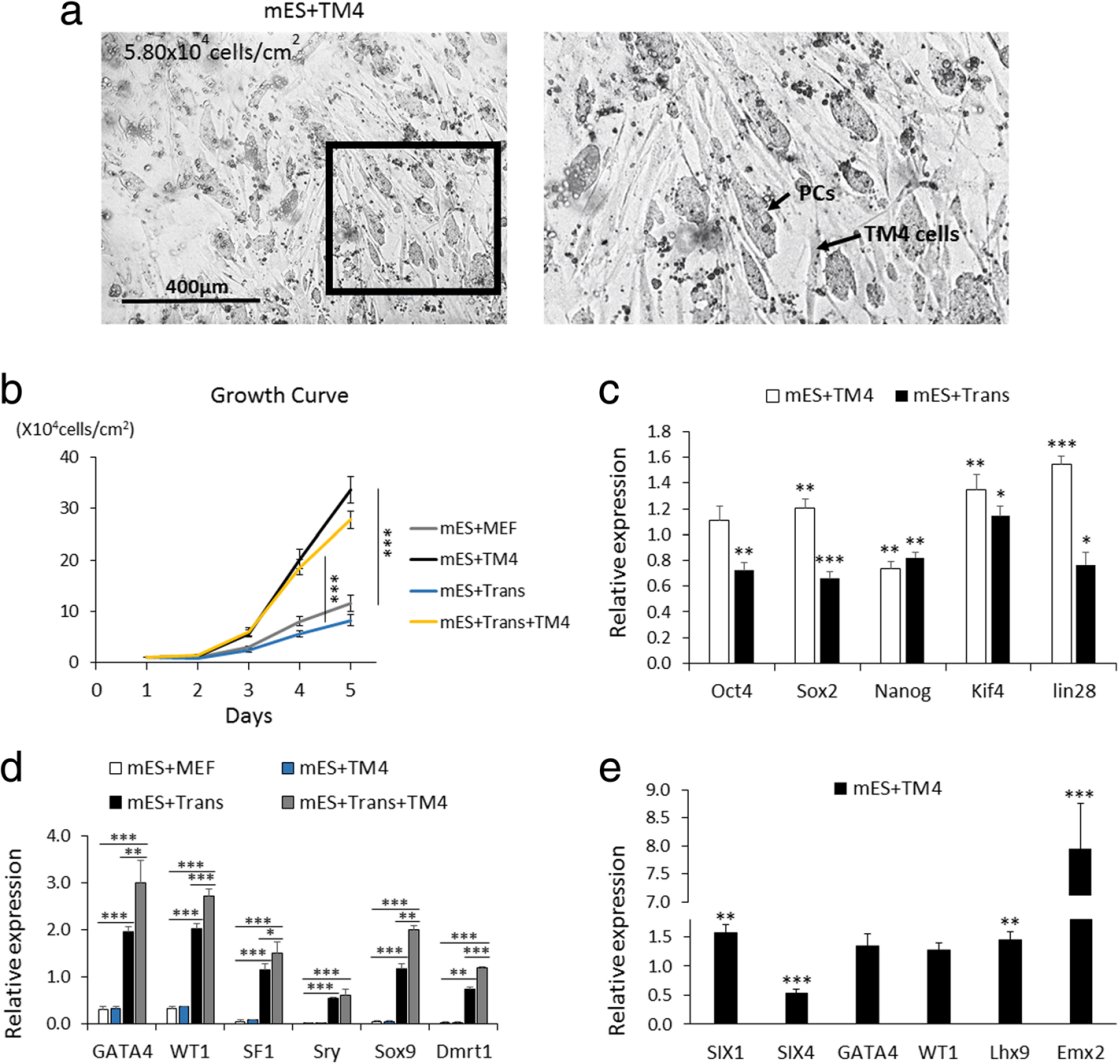
Recovering mES cells from cryopreservation with MEF or TM4 feeder. **a** At day 3, PCs were able to aggregate in group mES+TM4. Scale bar = 400 μm. Enlarged figure shows on the right. **b** Growth curves of mES cells in mES+MEF, mES+TM4, mES+Trans, and mES+Trans+TM4 groups in the first 5 days. Results were expressed as mean ± SD (*n* = 3 independent experiments). Asterisks indicate statistical significance calculated by one-way ANOVA method and labeled between groups mES+TM4 and mES+MEF at days 4 and 5. **c** Five pluripotency markers of cells were detected in groups mES+MEF, mES+TM4, and mES+Trans by qPCR at day 5. qPCR results were expressed relative to the expression in group mES+MEF (control group). Results were expressed as mean ± SD (*n* = 3 independent experiments). Asterisks indicate statistical significance of differences in the mean gene expression according to the control group (mES+MEF). **d** Transcriptional expression of lentiviral transduced factors in mES cells were detected in groups mES+MEF, mES+Trans, and mES+Trans+TM4. qPCR results show changes in introduced factors among different groups in gene expression relative to expression of β-actin in self-comparison. Results were expressed as mean ± SD (*n* = 3 independent experiments). Asterisks indicate statistical significance of differences in the mean gene expression between the indicated groups. **e** Transcriptional level of some early gonadogenesis markers in mES+TM4 were expressed relative to the expression in group mES+MEF (control group) at day 5. Results were expressed as mean ± SD (*n* = 3 independent experiments). Asterisks indicate statistical significance of differences in the mean gene expression according to the control group (mES+MEF). (*P value < 0.05, **P value < 0.01, ***P value < 0.001)

In a 5-day culture, compared to those with MEFs (group mES+MEF), mES cells co-cultured with TM4 cells (group mES+TM4) grew faster (Fig. 1b), were capable of forming pebble-like colonies (PCs) (Fig. 1a), and aggregated into PCs more quickly (Additional file 1: Figure S1A, B, C). Therefore, we recognized the TM4 cells as better feeder layers for promoting mES cell growth compared to MEF feeder layers via improving PC aggregation [50, 51]. Presumably, the improved growth of mES cells was due to the enhanced expression of Klf4 (Fig. 1c), as a factor working in self-renewal and proliferation of mES cells [52–55]. In addition, according to the results of the transcriptional level in comparison to group mES+MEF, mES cells in group mES+TM4 expressed four higher pluripotency biomarkers including Oct4, Sox2, Klf4, and lin28 (Fig. 1c) [56] and five developmental stage-specific markers including SIX1, GATA4, WT1, Lhx9, and Emx2 (Fig. 1e) [24]. Thus, the results indicate that the replacement of MEFs with TM4 cells improves the differentiation tendency of mES cells to develop into gonadal cells.

mES cells with lentiviral transduction of all the six factors, Sry, Sox9, SF1, WT1, GATA4, and Dmrt1 (mES+Trans) had a relatively less growth rate as compared to those without infection (group mES+MEF and mES+TM4) (Fig. 1b). Generally, lentiviral infection could harm mES cells, causing cell death, slow growth, and low expression of inserted factors. However, with TM4 feeder cells, the growth of mES cells (mES+Trans+TM4) was greatly improved as being much faster than groups mES+MEF and mES+Trans (Fig. 1b). Furthermore, the transcriptional expression level of the six introduced factors had a dramatic increase in mES cells cultured with TM4 feeder cells (mES+Trans+TM4) compared to those cultured with MEFs (mES+Trans) (Fig. 1d). Thus, as feeder layers, TM4 cells possess the ability to promote the expression of transduced factors in mES cells and to improve the cell growth against the harm caused by lentiviral transduction.

### The six factors WT1, Dmrt1, GATA4, SF1, Sry, and Sox9 play different stage-specific roles in inducing eSCs

**Fig. 2:**
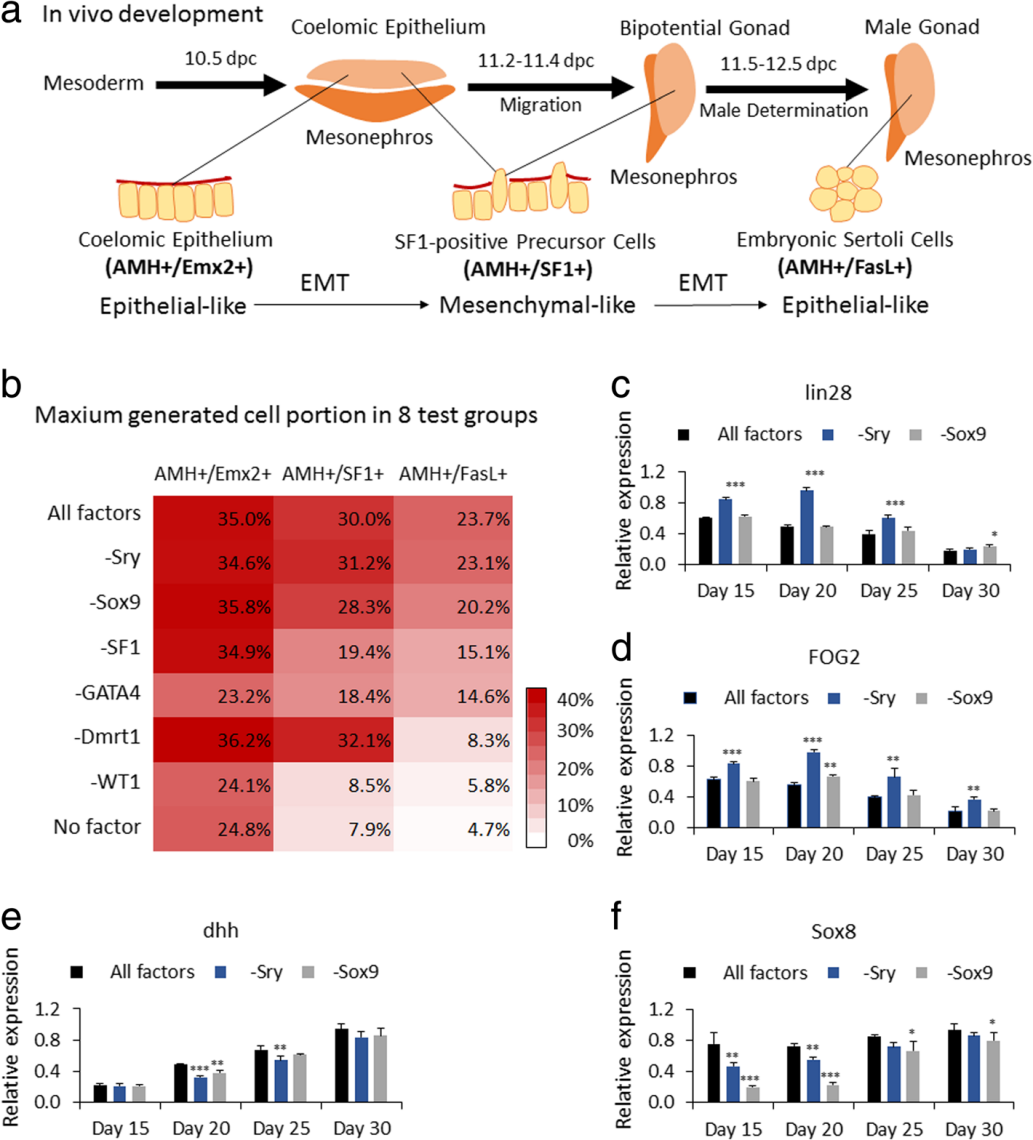
Development fate identification. **a** Stepwise development of progenitor cells to eSCs. **b** Heat map of stage-specific marker expression of different groups. In each transduction group, a different factor was removed from the pool of six factors. Group All factors was transduced with all the six factors. Group No factor was the control group (mES+MEF) without transfection. Group −Sry was the mES cells transduced with the six factors except for Sry. The rest were constructed in the same manner. Flow cytometry (FCM) with specific markers indicating different cell stages. AMH^+^/Emx2^+^ cells indicated coelomic epithelium. AMH^+^/SF1^+^ cells indicated SF1-positive precursor cells. AMH^+^/FasL^+^ cells indicated eSCs. Fluorescence antibody staining was performed after the samples had cell membrane perforation with Triton X-100. The results were expressed as mean value of the maximum positive cell portion of each test group in 35 days (*n* > 3 independent experiments). Gene expression levels of transcription factors **c** lin28, **d** FOG2, **e** dhh, and **f** Sox8 were detected by qPCR. Results show changes in gene expression relative to the highest expression in each group. **c**–**f** were expressed as mean ± SD (*n* = 3 independent experiments). Asterisks indicate statistical significance of differences in the mean of gene expression by one-way ANOVA according to the positive group (All factors) (*P value < 0.05, **P value < 0.01, ***P value < 0.001)

In vivo, eSCs specifically express genes Sry, Sox9, AMH, Sox8, and Dmrt1. Therefore, we selected these genes for genetic co-upregulation of mES cells to induce eSCs by lentiviral transduction. However, after 30 days of culturing, no obvious increase of eSCs was detected via IF or FCM (identification as FasL^+^/AMH^+^ cells). Therefore, we suspected that the improved generation of eSCs could involve a series of development stages including somatic cells of coelomic epithelium and SF1-positive precursor cells, then end up as eSCs (Fig. 2a).

Among the involved factors, WT1, GATA4, SF1, Lhx9, Emx2, Pod1, TIF1β, TIF2, and Insr facilitated the generation of somatic cells in coelomic epithelium [23–25, 46]; SIX1/4, FOG2/GATA4, Cbx2, Map3k4, and Gadd45g had high expression and complex interactions with other functional factors related to the development of progenitor cells of eSCs in bi-potential gonads [31]; Sry, Sox9, and Sox8 played an important role in male determination, and FGF9/FGFR2, PGD2, AMH, and Dmrt1 were highly expressed along the normal growth of eSCs [47]. To generate in vivo development leading to Sertoli cells, we selected WT1 and GATA4 for the generation of somatic cells in the coelomic epithelium. Some reports indicated the transcription factor GATA4 is expressed in the coelomic epithelium of the genital ridge, progressing in an anterior-to-posterior (A-P) direction, preceding an A-P wave of epithelial thickening [25], whereas WT1 fulfills its function in the gonads by regulating a set of target genes, including FOG2, SF1, Sry, and GATA4 [23, 57]. We speculated that the overexpression of WT1 and GATA4 can initiate a set of pathways, altering the fate of mES cells to gonadogenesis. AMH and Emx2 were selected to detect the generated coelomic epithelium. Steroidogenic factor 1 (SF1) was coded by the NR5A1 gene and helped in controlling the activity of several genes related to the development of the gonads [22, 58]; moreover, it is widely accepted that SF1-positive precursor cells in coelomic epithelium could be the main source of pre-Sertoli cells in the early urogenital ridge. It was important to test the developmental fate by regulating expression of SF1, and also the detection of SF1-positive precursor cells via the distinguishing expression degree of SF1 and AMH [9, 22]. Convincingly, Sry and Sox9 were found well concerned in male determination and development of early Sertoli cells [9, 45]. Accordingly, we also subjected Sry and Sox9 in a factor test and detected eSCs via AMH and FasL. Finally, some report analyses suggested the expression of Dmrt1 could be essential in maintaining male characteristic of eSCs [39, 47]. So the overexpression of Dmrt1 was included in the test. Therefore, six factors, WT1, GATA4, SF1, Sry, Sox9, and Dmrt1, were chosen to induce eSCs from mES cells; AMH, Emx2, SF1, and FasL were selected for developmental stage identification.

In a 35-day culture, a considerable number of induced eSCs (23.7% among the whole cell population) were generated with the transduction of all the six factors, Sry, Sox9, SF1, WT1, GATA4, and Dmrt1 (group All factors, i.e., mES+Trans) (Fig. 2b). To explore the function of each of the six factors in deriving eSCs, each transduction group had a different transcription factor removed. The population of coelomic epithelium (AMH^+^/Emx2^+^), SF1-positive precursor cells (AMH^+^/SF1^+^), and eSCs (AMH^+^/FasL^+^) were counted every week via FCM. We took the maximum value for evaluation. As shown in Fig. 2b, mES cells transduced without WT1 or Dmrt1 hardly generated eSCs (5.8% and 8.3%, respectively), which was very similar to the mES cell group with no upregulated factors (4.7%). The induced eSCs were greatly reduced without upregulation of GATA4 or SF1 (14.6% and 15.1%). Compared to the group with all factors (23.7%), eSC numbers were barely reduced in the absence of Sox9 or Sry (20.2% and 23.1%) (Additional file 1: Figure S2F). According to the results, mES cells without overexpression of WT1 (group −WT1) or GATA4 (group −GATA4) hardly developed into coelomic epithelium, which was similar to cells with no upregulated factors (group No factor, i.e., mES+MEF). Without upregulation of SF1, coelomic epithelium still formed while SF1-positive precursor cells were not obviously promoted. Overexpression deficiency of Sry (group −Sry) and Sox9 (group −Sox9) did not show obvious difference in generating coelomic epithelium, SF1-positive precursor cells, and eSCs compared to group All factors. Notably, although the generation of coelomic epithelium and SF1-positive precursor cells had been promoted, eSCs could hardly be detected without transcriptional activation of Dmrt1 (group −Dmrt1). We conjectured that eSCs were generated in this group at day 30; however, without activation of Dmrt1, induced eSCs could not maintain male development and lost characteristic expression of male-specific biomarkers including FasL.

The results in Fig. 2b indicated that Sry or Sox9 could have no indispensable role in inducing eSCs. However, we still speculated these two factors had a contribution in some way. So we detected lin28 (mainly expressed in some stem cells), FOG2 (highly expressed in coelomic epithelium and gonads), dhh (highly expressed in male gonads and eSCs), and Sox8 (expressed along the development of eSCs) in a transcriptional level every 5 days (Fig. 2c–f). The data showed no overexpression of Sry; these mES cells (−Sry) highly expressed lin28 and FOG2 for a longer period and upregulated dhh and Sox8 which were later compared to those in group All factors. This result indicates that the generation of eSCs was delayed without lentiviral upregulation of Sry, being in accord with the cell population curve (Additional file 1: Figure S2G). In addition, without enhanced Sox9, the growth of eSCs (−Sox9) was delayed and the maximum population decreased by 3.5% (mean value) and had a statistical difference (*P* value = 0.004) (Additional file 1: Figure S2F, G). These evidences indicated that the overexpression of Sry and Sox9 had a certain significance.

All these evidences indicated that the function of individual factors was very likely to play different stage-specific roles in the derivation of eSCs and concealed some complicated interactions.

### Cellular morphology identification of the derivation process of Sertoli cells

Cell colonies of mES cells in vitro generally form into PCs. Morphological observation could deliver evidence to identify the presented cell type, characteristic, and developmental stage.

**Fig. 3:**
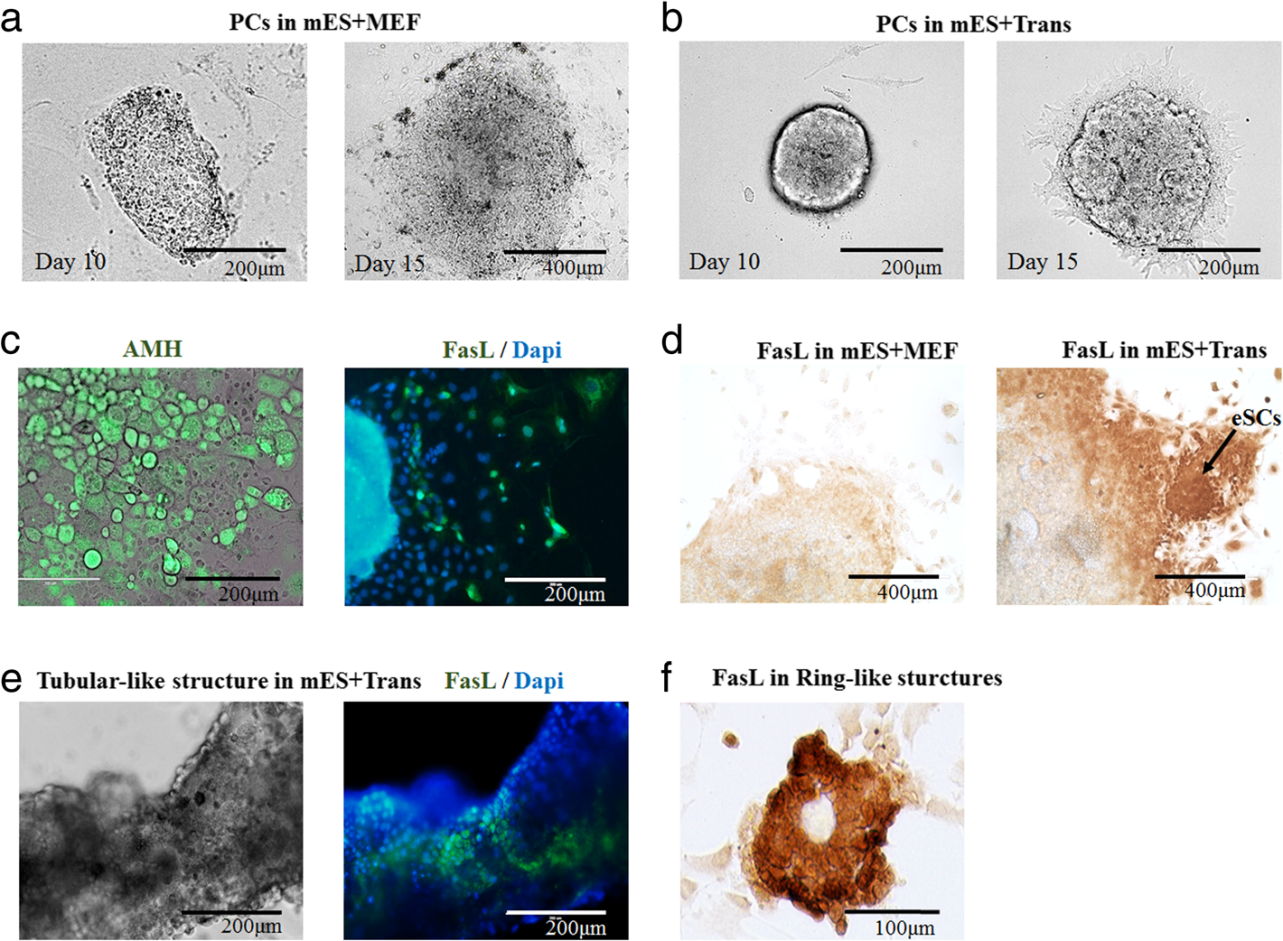
Observing the induction process with FCM, IF, ICC, and qPCR. Optical micrographs **a** and **b** show the morphological characteristics of the PCs of mES+MEF and mES+Trans groups. In **a**, scale bar = 200 μm and 400 μm for the left and right panels, respectively. In **b**, scale bar = 200 μm. **c** Optical micrographs and IF were performed in group mES+Trans at day 20. Brightfield and fluorescence of AMH (green) images were merged on the left. Fluorescence of FasL (green) and Dapi (blue) merged imaging on the right. Scale bar = 200 μm. **d** ICC was performed with FasL antibody in groups mES+MEF and mES+Trans at day 30. Dark brown color shows the positive cells. Pale brown and achromatic colors show the negative cells. Scale bar = 400 μm. **e** At day 35 in group mES+Trans, tubular-like structure colonies were observed. Results show in optical micrographs on the left, and fluorescence merging images FasL (green) and Dapi (blue) on the right. Scale bar = 200 μm. **f** At day 30, FasL-positive cells formed ring-like structures in group mES+Trans. Scale bar = 100 μm

Under an optical microscope, PCs in the group mES+Trans remained much more aggregated than those without six introduced factors (group mES+MEF) in the first 15 days (Fig. 3a, b). At day 20, by IF, a large population of AMH^+^ cells displayed epithelial-like cellular morphology, and there was found a small number of FasL^+^ cells scattering around PCs, being speculated as incipient generated eSCs (Fig. 3c). At day 30, according to the ICC result, FasL^+^ cells (dark brown) grew around PCs in group mES+Trans and some of them formed into colonies (Fig. 3d). These FasL^+^ cells were most likely to be eSCs. In comparison, most of the cells showed to be FasL-negative (pale brown) in the control group (mES+MEF).

**Fig. 4:**
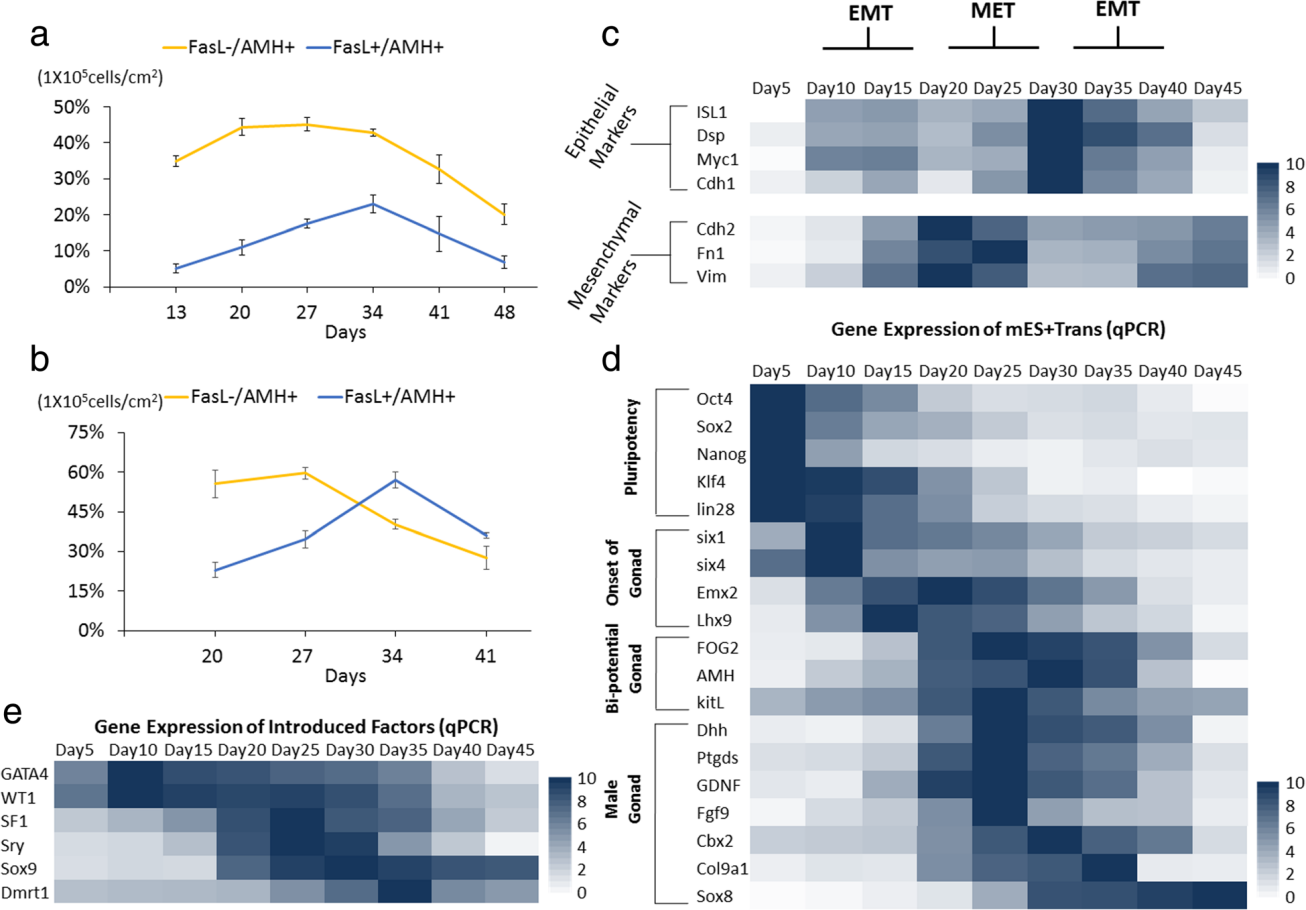
Identification of morphological changes with biomarkers, external features, and FCM. The cell portion of FasL^−^/AMH^+^ and FasL^+^/AMH^+^ cells in group mES+Trans were detected via FCM. The result of the whole cell population of mES+Trans between 13 and 48 days is shown in **a**. The result of the cells removed of PCs between 13 and 34 days is shown in **b**. Tests were performed every 7 days and expressed as mean ± SD (*n* = 3 independent experiments). **c** Identification of the transcriptional level of epithelial markers and mesenchymal markers reveals the timing of morphological transformation (MET or EMT) between 5 and 45 days. qPCR was performed every 5 days and expressed relative to the highest expression among each marker. qPCR results took the mean value and are shown in the heat map (*n* = 3 independent experiments). MET mesenchymal to epithelial transformation. EMT epithelial to mesenchymal transformation. Heat map indicates the gene expression levels of major transcription factors in group mES+Trans for **d** pluripotency, onset of gonads, bi-potential gonads, and male gonads, and for **e** introduced factors. qPCR results show changes in gene expression relative to the highest expression in each marker. Results were expressed as mean and transformed into heat map (*n* > 2)

eSCs in vivo have a tendency to form tubular-like structures which later consist seminiferous tubules in the male genital ridge. After a 40-day culture, under an immuno-electron microscope, a tubular-like structure was displayed according to the blue fluorescent cell nucleus stained by Dapi (Fig. 3e). However, only a part of these cells indicated to be FasL-positive (green fluorescence). According to the result of FCM identification, a good number of cells lost FasL expression after day 34 (Fig. 4a). Thus, the cells other than FasL^+^ cells in the tubular-like structure were speculated to be other somatic cells or eSCs losing the expression capacity of FasL due to the development or culture condition. In addition, when cultured in low cell confluence, FasL^+^ cells formed into ring-like structures at day 30 as a normal characteristic of eSCs (Fig. 3f).

### Development parameters in derivation of Sertoli cells

To analyze the interrelation between the progenitor cells and generation of eSCs, FasL^−^/AMH^+^ cells were identified as a complex cell population including coelomic epithelium and some kinds of somatic cells in the gonads; FasL^+^/AMH^+^ cells were identified as eSCs. In FCM of group mES+Trans, FasL^−^/AMH^+^ cells kept increasing during the first 20 days of induction, and then dropped afterwards (Fig. 4a). FasL^+^/AMH^+^ cells gradually grew in quantity until day 34, followed by an immediate reduction. The period of increased cell portion of eSCs just met the period of decreased portion of FasL^−^/AMH^+^ cells. For a more accurate result, the PCs were removed by mechanical isolation (pipette blow, cell scrapers, and lifters). While only the differentiated cells spreading around were left, a clear growth trend of FasL^−^/AMH^+^ cells and FasL^+^/AMH^+^ cells was observed (Fig. 4b). Between 20 and 27 days, the cell portion of FasL^−^/AMH^+^ cells rapidly declined when eSCs had an obvious increase and reached 60% at day 27. Notably, the SUM of these AMH^+^ cells occupied most cell population among these differentiated cells between 13 and 34 days (78.57%, 94.13%, 97.1%, 63.64%). These results implied that the progenitor cells of eSCs could have migrated away from PCs and gradually developed into eSCs. This conclusion accorded with the result shown in Fig. 3c that many AMH^+^ cells grew around or away from PCs and some FasL^+^ cells separately appeared among the somatic cells outside of the PCs. It is interesting that the progenitor cells of eSCs in vivo also have a stage of migration from the coelomic epithelium to bi-potential gonads [6].

Single cell morphological changes provide significant information. As progenitor cells of eSCs, somatic cells in the coelomic epithelium were epithelial-like, SF1-positive precursor cells were mesenchymal-like, and eSCs were epithelial-like (Fig. 2a). In an in vitro environment, immature and mature Sertoli cells presented a mesenchymal-like characteristic. Via epithelial markers and mesenchymal markers, the first EMT was speculated to be happening between 10 and 20 days; MET happened between 20 and 30 days, and the second EMT happened between 30 and 40 days (Fig. 4c). These results accorded with the sudden decline of FasL^+^/AMH^+^ cells (Fig. 4b), indicating the expression deficiency of FasL could result from the development of eSCs into immature Sertoli cells or the morphological changes from epithelial-like to mesenchymal-like.

In transcription expression of the major markers, gonad development was ready at day 5 and initiated at day 10. Gonad-related marker genes were significantly increased after day 15 (Fig. 4d). At day 20, male-determining genes and male gonad-specific marker genes were simultaneously activated. These results clarify the exact periods of different developmental stages. Transcriptional detection of the six introduced factors by qPCR showed their changes along the development (Fig. 4e). They greatly enhanced the expression between 10 and 35 days due to endogenous gene expression. However, after day 40, most of the male gonad markers greatly declined except Sox8 which might have improved by the overexpression of Sox9. In joint analysis of the cell population curve (Fig. 4a) and transcription expression (Fig. 4d), we speculated that the culture condition could be inadequate for maintenance and growth of generated eSCs, or these eSCs had developed into immature Sertoli cells with altered transcription expression.

## Discussion

### A novel and potentially efficient approach to deliver eSCs

In this work, a large population of eSCs was successfully induced from mES cells via overexpression of six factors, Sry, Sox9, SF1, WT1, GATA4, and Dmrt1. Here, we established a potential efficient method to deliver eSCs occupying 24% of the whole cell population (1 × 10^5^ cells/cm^2^). With PCs removed, the proportion of eSCs reached 60%. In comparison to other inducing methods [19, 20], in this approach while improving the population of Sertoli cells from human embryonic stem cells by reducing the size of the cultured cell colonies, the FSHR-positive cells (human Sertoli cells) reached 30–35% in the whole cell population [19]. In the previous study, the method generating induced embryonic Sertoli-like cells transdifferentiated from mouse fibroblasts; although there was no direct data of the cell proportion of generated Sertoli-like cells, the induced embryonic Sertoli-like cells were speculated to occupy the main cell population and reached a relatively high purity for about 10 days induction [20]. Because the mouse fibroblasts are mitosis-inactivated and these Sertoli-like cells showed rather high proliferative capability, thus, the inducing approach we built did not have obvious advantage in inducing efficiency; however, our approach is easy to operate and performs better to induce the in vivo development of Sertoli cells, which could be beneficial for the developmental mechanism study in gonadogenesis and clinical application.

However, there were still some barriers in the application of induced eSCs derived from mES cells. As AMH is a secreted factor and Sox9 is nuclear, we need to isolate eSCs by flow cytometry sorting (FACS) according to FasL antibody alone. In the survey sampling of these FasL^+^ cells, samples were treated with Triton X-100, with 90% (± 3%) AMH^+^ and 50% (± 10%) Sox9^+^ in FCM identification (Additional file 1: Figure S3A, B, C). Thus, we considered these cells as mainly eSCs and performed a mice test with them (Additional file 1: Figure S4A, B, C, D). However, we realized that there was a flaw in the experiment design. Unlike the approach of reprogramming fibroblast, we cannot clarify the cell types in these FasL^+^ cell populations besides eSCs, which were present as AMH^−^ (about 10%) or Sox9^−^ (about 50%). Furthermore, the mice test did not include cyto-dynamics (BrdU pulse or H2B-GFP), living cell identification, and further systematic biomarker identification. All these deficiencies would be improved, and therefore further research work is required to accomplish this task.

### Modeling a roadmap for the derivation of eSCs in vitro

**Fig. 5:**
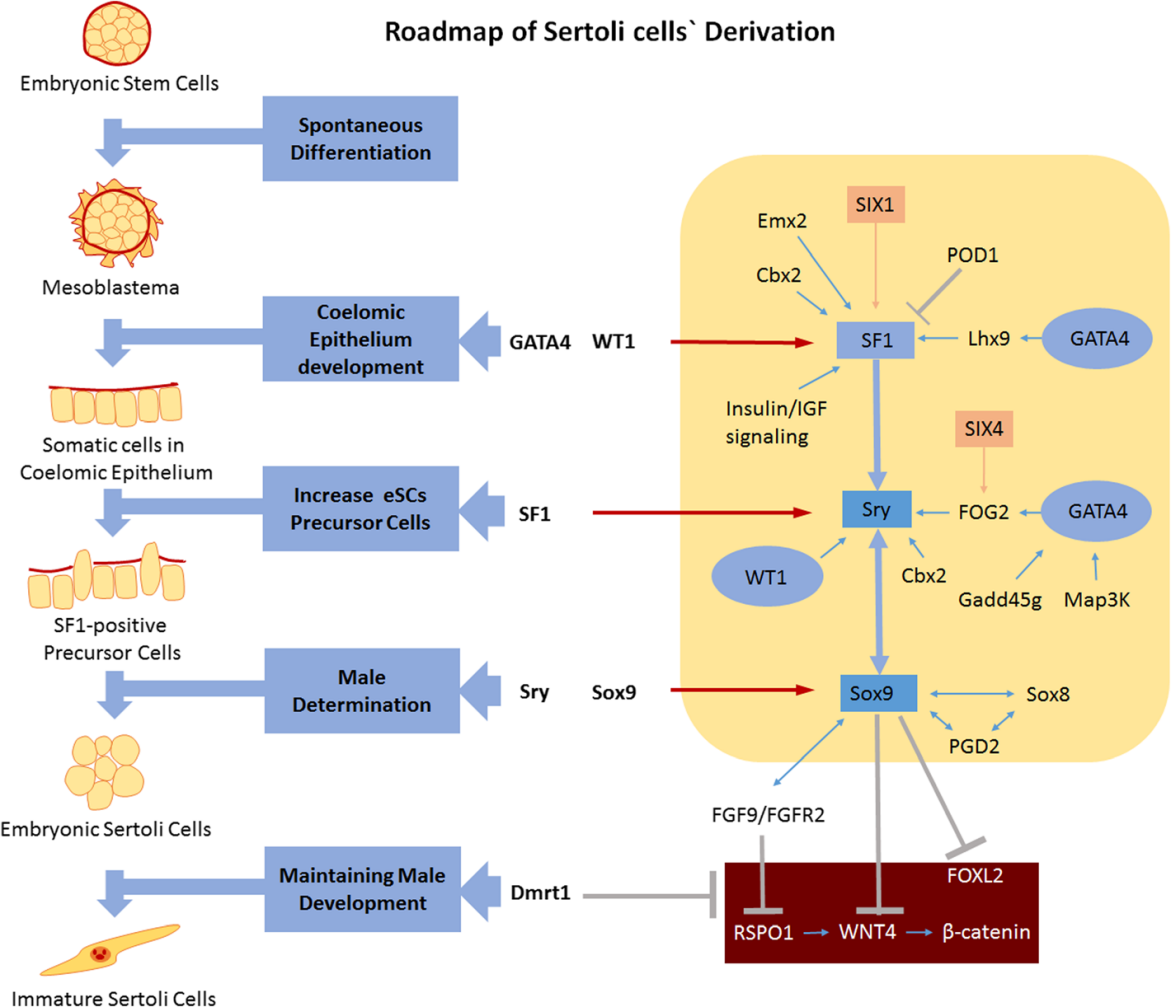
Differentiation roadmap of generating Sertoli cells. A speculated stepwise derivation process of the mouse Sertoli cell including relevant molecular mechanisms. GATA4 and WT1 promote the generation of coelomic epithelium. SF1 increases the direct precursor cells of eSCs. Sry and Sox9 improve male determination. Dmrt1 maintains the male development of eSCs and suppresses female determination with Sox9

In this work, the functions of the six transcriptional factors were determined during the derivation of eSCs. Previously, the functions of WT1, GATA4, SF1, Sry, Sox9, and Dmrt1 were separately explored. Here, we established an interconnection between the combined actions of the six inducing factors at different developmental stages (Fig. 5).

As a first step, mES cells grow and spontaneously differentiate into three germ layers under an environment without LIF. In differentiated triploblasts, the coelomic epithelium developed from the mesoblastema via GATA4 and WT1. After that, somatic cells in the coelomic epithelium develops into SF1-positive precursor cells with the activation of SF1 enhanced by GATA4, WT1, and additional relevant factors expressed in the coelomic epithelium including Cbx2, Emx2, SIX1, Lhx9, and insulin/IGF. Then the fate of SF1-positive precursor cells diverges due to sex determination. Under the influence of Cbx2, FOG2, WT1, and SF1, Sry is activated and eSCs are derived from SF1-positive precursor cells. Increased expression of Sox9 in eSCs strengthens male determination and positively regulates the expression of FGF9/FAFR2. Via the combined action of Sox9, FGF9/FGFR2, and Dmrt1, female determining signals including RSPO1, WNT4, β-catenin, and FOXL2 are inhibited, which guarantees the development of eSCs into immature Sertoli cells. Finally, these immature Sertoli cells develop into mature Sertoli cells in the testicular environment.

The roadmap model of Sertoli cell derivation would be beneficial for revealing developmental mechanism and sex determination. To further improve the development mechanism model, more information and data could be achieved as follows: (1) accurate identification of all parts of the cell population in the different development stages from mES cells to eSCs via overexpression of the six inducing factors (by FCM or IF), (2) comprehensive detection of the cell model (by microarray or protein array), (3) determination of other functional factors interacting with the known factors, (4) altering the development fate of mES cells by expression deficiency or overexpression of potential functional factors besides the six inducing factors, and (5) since WT1, GATA4, and SF1 have positive influence on the expression of Sry and Sox9, and Sry and Sox9 have positive feedback between each other, thus, the individual factor investigation of Sry and Sox9 should be rigorously designed while involving complex gene silence array of all the 6 factors, (6) given the known functions of Dmrt1, we speculate that eSCs were successfully induced in group –Dmrt; however, they lost some biomarkers including FasL and AMH, or may even have undergone female conversion without genetic upregulation of Dmrt1 [39, 40, 59, 60]. If so, this attaches new importance to Dmrt1 and draws forth a meaningful survey on the regulation of Dmrt1 in the inducing pathway of eSCs.

## Conclusion

In this study, we determined the six core inducing factors and modeled a differentiation roadmap of mouse embryonic stem cells into embryonic Sertoli cells. By the overexpression of Sry, Sox9, SF1, WT1, GATA4, and Dmrt1, the large amounts of embryonic Sertoli cells (eSCs) were successfully induced from mouse embryonic stem (mES) cells. Moreover, we suggested that the six factors played different stage-specific roles in inducing eSCs. These new findings prospect a potential way for further revealing the mechanisms of male gonadal development and providing potential tools for tracking male fetal reproductive disease. In short, we present a novel and efficient approach to deliver embryonic Sertoli cells from mouse embryonic stem cells. Conclusively, the systematically conducted study presents an efficient approach that will surely help in future to cure the reproductive diseases.

## Additional file

Additional file 1: Experimental methods. (DOCX 4120 kb)

## Abbreviations

ANOVA: Analysis of variance
A-P: Anterior-to-posterior
BSA: Bovine serum albumin
EMT: Epithelial-mesenchymal transformation
eSCs: Embryonic Sertoli cells
FBS: Fetal calf serum
FCM: Flow cytometry
ICC: Immunocytochemistry
IF: Immunofluorescence
LIF: Leukemia inhibitory factor
MEFs: Mouse embryo fibroblasts
mES: Mouse embryonic stem
MET: Mesenchymal-epithelial transformation
NEAA: Non-essential amino acids
PCs: Pebble-like colonies
qPCR: Quantitative real-time polymerase chain reaction
SD: Standard deviation
SF1: Steroidogenic factor 1
SPSS: Statistical Package for Science and Society

## References

[1] Li Y, Xue W, Tian X, Ding X, Tian P, Feng X, Song Y, Luo X, Liu H, Wang X, Ding C (2011). Improved survival and function of rat cryopreserved islets by coculture with sertoli cells. Artif Organs.

[2] Miryounesi M, Nayernia K, Dianatpour M, Mansouri F, Modarressi MH (2013). Co-culture of mouse embryonic stem cells with Sertoli cells promote in vitro generation of germ cells. Iran J Basic Med Sci.

[3] Tian H, Guo M, Zhuang Y, Chu J, Zhang S (2014). Enhanced proliferation of bone marrow mesenchymal stem cells by co-culture with TM4 mouse Sertoli cells: involvement of the EGF/PI3K/AKT pathway. Mol Cell Biochem.

[4] Skinner MK, Griswold MD (2005). Sertoli Cell Biology.

[5] Hiramatsu R, Matoba S, Kanai-Azuma M, Tsunekawa N, Katoh-Fukui Y, Kurohmaru M, Morohashi K, Wilhelm D, Koopman P, Kanai Y (2009). A critical time window of Sry action in gonadal sex determination in mice. Development.

[6] Barrionuevo F, Burgos M, Jimenez R (2011). Origin and function of embryonic Sertoli cells. Biomol Concepts.

[7] Nel-Themaat L, Jang CW, Stewart MD, Akiyama H, Viger RS, Behringer RR (2011). Sertoli cell behaviors in developing testis cords and postnatal seminiferous tubules of the mouse. Biol Reprod.

[8] Chojnacka K, Zarzycka M, Mruk DD (2016). Biology of the Sertoli cell in the fetal, pubertal, and adult mammalian testis. Results Probl Cell Differ.

[9] Piprek RP, Kloc M, Kubiak JZ (2016). Early development of the gonads: origin and differentiation of the somatic cells of the genital ridges. Results Probl Cell Differ.

[10] Sun Z, Nie Q, Zhang L, Niu R, Wang J, Wang S (2017). Fluoride reduced the immune privileged function of mouse Sertoli cells via the regulation of Fas/FasL system. Chemosphere.

[11] Shi B, Deng L, Shi X, Dai S, Zhang H, Wang Y, Bi J, Guo M (2012). The enhancement of neural stem cell survival and growth by coculturing with expanded Sertoli cells in vitro. Biotechnol Prog.

[12] Deng L, Shi B, Zhuang Y, Chu J, Shi X, Zhang S, Guo M (2014). Performance and mechanism of neuroleukin in the growth and survival of sertoli cell-induced neurons in a coculture system. Cell Transplant.

[13] Zhang F, Lu M, Liu H, Ren T, Miao Y, Wang J (2016). Sertoli cells promote proliferation of bone marrow-derived mesenchymal stem cells in co-culture. Indian J Exp Biol.

[14] Fan P, He L, Pu D, Lv X, Zhou W, Sun Y, Hu N (2011). Testicular Sertoli cells influence the proliferation and immunogenicity of co-cultured endothelial cells. Biochem Biophys Res Commun.

[15] Zeller P, Legendre A, Jacques S, Fleury MJ, Gilard F, Tcherkez G, Leclerc E (2017). Hepatocytes cocultured with Sertoli cells in bioreactor favors Sertoli barrier tightness in rat. J Appl Toxicol.

[16] Griswold MD. 50 years of spermatogenesis: Sertoli cells and their interactions with germ cells. Biol Reprod. 2018. 10.1093/biolre/ioy027 PMC7328471 29462262

[17] Baazm M, Mashayekhi FJ, Babaie S, Bayat P, Beyer C, Zendedel A (2017). Effects of different Sertoli cell types on the maintenance of adult spermatogonial stem cells in vitro. In Vitro Cell Dev Biol Anim.

[18] Chui K, Trivedi A, Cheng CY, Cherbavaz DB, Dazin PF, Huynh AL, Mitchell JB, Rabinovich GA, Noble-Haeusslein LJ, John CM (2011). Characterization and functionality of proliferative human Sertoli cells. Cell Transplant.

[19] Bucay N, Yebra M, Cirulli V, Afrikanova I, Kaido T, Hayek A, Montgomery AM (2009). A novel approach for the derivation of putative primordial germ cells and sertoli cells from human embryonic stem cells. Stem Cells.

[20] Buganim Y, Itskovich E, Hu YC, Cheng AW, Ganz K, Sarkar S, Fu D, Welstead GG, Page DC, Jaenisch R (2012). Direct reprogramming of fibroblasts into embryonic Sertoli-like cells by defined factors. Cell Stem Cell.

[21] Karl J, Capel B (1998). Sertoli cells of the mouse testis originate from the coelomic epithelium. Dev Biol.

[22] Sekido R, Lovell-Badge R (2008). Sex determination involves synergistic action of SRY and SF1 on a specific Sox9 enhancer. Nature.

[23] Hammes A, Guo JK, Lutsch G, Leheste JR, Landrock D, Ziegler U, Gubler MC, Schedl A (2001). Two splice variants of the Wilms’ tumor 1 gene have distinct functions during sex determination and nephron formation. Cell.

[24] Tevosian SG, Albrecht KH, Crispino JD, Fujiwara Y, Eicher EM, Orkin SH (2002). Gonadal differentiation, sex determination and normal Sry expression in mice require direct interaction between transcription partners GATA4 and FOG2. Development.

[25] Hu YC, Okumura LM, Page DC (2013). Gata4 is required for formation of the genital ridge in mice. PLoS Genet.

[26] Birk OS, Casiano DE, Wassif CA, Cogliati T, Zhao L, Zhao Y, Grinberg A, Huang S, Kreidberg JA, Parker KL, Porter FD, Westphal H (2000). The LIM homeobox gene Lhx9 is essential for mouse gonad formation. Nature.

[27] Kusaka M, Katoh-Fukui Y, Ogawa H, Miyabayashi K, Baba T, Shima Y, Sugiyama N, Sugimoto Y, Okuno Y, Kodama R, Iizuka-Kogo A, Senda T, Sasaoka T, Kitamura K, Aizawa S, Morohashi K (2010). Abnormal epithelial cell polarity and ectopic epidermal growth factor receptor (EGFR) expression induced in Emx2 KO embryonic gonads. Endocrinology.

[28] Fujimoto Y, Tanaka SS, Yamaguchi YL, Kobayashi H, Kuroki S, Tachibana M, Shinomura M, Kanai Y, Morohashi K, Kawakami K, Nishinakamura R (2013). Homeoproteins Six1 and Six4 regulate male sex determination and mouse gonadal development. Dev Cell.

[29] Katoh-Fukui Y, Miyabayashi K, Komatsu T, Owaki A, Baba T, Shima Y, Kidokoro T, Kanai Y, Schedl A, Wilhelm D, Koopman P, Okuno Y, Morohashi K (2012). Cbx2, a polycomb group gene, is required for Sry gene expression in mice. Endocrinology.

[30] Bogani D, Siggers P, Brixey R, Warr N, Beddow S, Edwards J, Williams D, Wilhelm D, Koopman P, Flavell RA, Chi H, Ostrer H, Wells S, Cheeseman M, Greenfield A (2009). Loss of mitogen-activated protein kinase kinase kinase 4 (MAP3K4) reveals a requirement for MAPK signalling in mouse sex determination. PLoS Biol.

[31] Warr N, Siggers P, Carre GA, Bogani D, Brixey R, Akiyoshi M, Tachibana M, Teboul L, Wells S, Sanderson J, Greenfield A (2014). Transgenic expression of Map3k4 rescues T-associated sex reversal (Tas) in mice. Hum Mol Genet.

[32] Warr N, Carre GA, Siggers P, Faleato JV, Brixey R, Pope M, Bogani D, Childers M, Wells S, Scudamore CL, Tedesco M, del Barco Barrantes I, Nebreda AR, Trainor PA, Greenfield A (2012). Gadd45gamma and Map3k4 interactions regulate mouse testis determination via p38 MAPK-mediated control of Sry expression. Dev Cell.

[33] Bouma GJ, Washburn LL, Albrecht KH, Eicher EM (2007). Correct dosage of Fog2 and Gata4 transcription factors is critical for fetal testis development in mice. Proc Natl Acad Sci U S A.

[34] Barrionuevo FJ, A Hurtado, GJ Kim, FM Real and M Bakkali. (2016). Sox9 and Sox8 protect the adult testis from male-to-female genetic reprogramming and complete degeneration. 5. 10.7554/eLife.15635 PMC4945155 27328324

[35] Colvin JS, Green RP, Schmahl J, Capel B, Ornitz DM (2001). Male-to-female sex reversal in mice lacking fibroblast growth factor 9. Cell.

[36] Kim Y, Kobayashi A, Sekido R, DiNapoli L, Brennan J, Chaboissier MC, Poulat F, Behringer RR, Lovell-Badge R, Capel B (2006). Fgf9 and Wnt4 act as antagonistic signals to regulate mammalian sex determination. PLoS Biol.

[37] Moniot B, Declosmenil F, Barrionuevo F, Scherer G, Aritake K, Malki S, Marzi L, Cohen-Solal A, Georg I, Klattig J, Englert C, Kim Y, Capel B, Eguchi N, Urade Y, Boizet-Bonhoure B, Poulat F (2009). The PGD2 pathway, independently of FGF9, amplifies SOX9 activity in Sertoli cells during male sexual differentiation. Development.

[38] Blagosklonova O, Joanne C, Roux C, Bittard H, Fellmann F, Bresson JL (2002). Absence of anti-Mullerian hormone (AMH) and M2A immunoreactivities in Sertoli cell-only syndrome and maturation arrest with and without AZF microdeletions. Hum Reprod.

[39] Matson CK, Murphy MW, Sarver AL, Griswold MD, Bardwell VJ, Zarkower D (2011). DMRT1 prevents female reprogramming in the postnatal mammalian testis. Nature.

[40] Lindeman RE, Gearhart MD, Minkina A, Krentz AD, Bardwell VJ, Zarkower D (2015). Sexual cell-fate reprogramming in the ovary by DMRT1. Curr Biol.

[41] Lau YF, Li Y (2009). The human and mouse sex-determining SRY genes repress the Rspol/beta-catenin signaling. J Genet Genomics.

[42] Jameson SA, Lin YT, Capel B (2012). Testis development requires the repression of Wnt4 by Fgf signaling. Dev Biol.

[43] Meeks JJ, Weiss J, Jameson JL (2003). Dax1 is required for testis determination. Nat Genet.

[44] Bertho S, Pasquier J, Pan Q, Le Trionnaire G, Bobe J, Postlethwait JH, Pailhoux E, Schartl M, Herpin A, Guiguen Y (2016). Foxl2 and its relatives are evolutionary conserved players in gonadal sex differentiation. Sex Dev.

[45] Li Y, Zheng M, Lau YF (2014). The sex-determining factors SRY and SOX9 regulate similar target genes and promote testis cord formation during testicular differentiation. Cell Rep.

[46] Zhang L, Chen M, Wen Q, Li Y, Wang Y, Wang Y, Qin Y, Cui X, Yang L, Huff V, Gao F (2015). Reprogramming of Sertoli cells to fetal-like Leydig cells by Wt1 ablation. Proc Natl Acad Sci U S A.

[47] Zhao L, Svingen T, Ng ET, Koopman P (2015). Female-to-male sex reversal in mice caused by transgenic overexpression of Dmrt1. Development.

[48] Gunes SO, Metin Mahmutoglu A, Agarwal A (2016). Genetic and epigenetic effects in sex determination. Birth Defects Res C Embryo Today.

[49] Wiles MV (1993). Embryonic stem cell differentiation in vitro. Methods Enzymol.

[50] Desbaillets I, Ziegler U, Groscurth P, Gassmann M (2000). Embryoid bodies: an in vitro model of mouse embryogenesis. Exp Physiol.

[51] Kurosawa H (2007). Methods for inducing embryoid body formation: in vitro differentiation system of embryonic stem cells. J Biosci Bioeng.

[52] Takahashi K, Yamanaka S (2006). Induction of pluripotent stem cells from mouse embryonic and adult fibroblast cultures by defined factors. Cell.

[53] Wernig M, Meissner A, Foreman R, Brambrink T, Ku M, Hochedlinger K, Bernstein BE, Jaenisch R (2007). In vitro reprogramming of fibroblasts into a pluripotent ES-cell-like state. Nature.

[54] Stadtfeld M, Nagaya M, Utikal J, Weir G, Hochedlinger K (2008). Induced pluripotent stem cells generated without viral integration. Science.

[55] Papp B, Plath K (2011). Reprogramming to pluripotency: stepwise resetting of the epigenetic landscape. Cell Res.

[56] De Los AA, Ferrari F, Xi R, Fujiwara Y, Benvenisty N, Deng H, Hochedlinger K, Jaenisch R, Lee S, Leitch HG, Lensch MW, Lujan E, Pei D, Rossant J, Wernig M, Park PJ, Daley GQ (2015). Hallmarks of pluripotency. Nature.

[57] Klattig J, Sierig R, Kruspe D, Makki MS, Englert C (2007). WT1-mediated gene regulation in early urogenital ridge development. Sex Dev.

[58] Ludbrook LM, Bernard P, Bagheri-Fam S, Ryan J, Sekido R, Wilhelm D, Lovell-Badge R, Harley VR (2012). Excess DAX1 leads to XY ovotesticular disorder of sex development (DSD) in mice by inhibiting steroidogenic factor-1 (SF1) activation of the testis enhancer of SRY-box-9 (Sox9). Endocrinology.

[59] Smith CA, Katz M, Sinclair AH (2003). DMRT1 is upregulated in the gonads during female-to-male sex reversal in ZW chicken embryos. Biol Reprod.

[60] Minkina A, Matson CK, Lindeman RE, Ghyselinck NB, Bardwell VJ, Zarkower D (2014). DMRT1 protects male gonadal cells from retinoid-dependent sexual transdifferentiation. Dev Cell.

