# Supplementary material for: Differentiation roadmap of embryonic Sertoli cells derived from mouse embryonic stem cells
Source: Stem Cell Res Ther. 2019 Mar 8;10:81. doi: 10.1186/s13287-019-1180-6 (PMC6408820; doi:10.1186/s13287-019-1180-6)
Supplement: Supplementary file 1 — Experimental methods. (DOCX 4120 kb) [file 13287_2019_1180_MOESM1_ESM.docx]

**Additional file 1**

**EXPERIMENTAL METHODS**

**Injection of Induced eSCs into testes**

Induced eSCs were sorted as FasL^+^ cells from the mES+Trans groups with CD178 (Fas ligand) antibody (eBioscience, USA) at the 30th day (**Table S6**). After washing with culture medium without serum, eSCs were re-suspended in buffer C, and mixed with PKH26 staining solution (Sigma Aldrich, USA) according to manufacturer`s instructions. The mixture was incubated for 2–5 min at 25°C. Then the reaction was ceased by adding an equal volume of 1.0% BSA, and the fluorescence staining of eSCs was observed under an EVOS FL Auto imaging system (Life Technologies, USA).

To anesthetize test mice, mice were injected with 1.0% pelltobarbitalum patricum (10mg/mL) according to each individual mouse (45 mg dose/kg weight). After confirming the unconscious, the abdominal cavities of ten comatose 2-week-old mice were surgically opened to expose the testes. Along with the testicular efferent ductules, stained eSCs were injected by injection syringes [1][2].

After one week, mice were anesthetized in sealed containers filled with diethyl ether gas. After losing consciousness, test mice were executed by cervical dislocation. After that, their abdominal cavities were surgically opened, and testicles were removed with tweezers and dissected with scalpers to reveal the seminiferous tubules for biopsy use and observation under an EVOS FL Auto imaging system (Life Technologies, USA). For IHC-P (paraffin) identification, seminiferous tubules were cut into paraffin sections and stained with DDX4 and PGP9.5 antibodies (Invitrogen, USA).

For mouse experiments, total ten male mice were operated on. Two were died right after surgery due to drug toxicity of anesthesia, and three were died within one week due to bacterial infection. All experimental operations were performed in accordance with the laws, regulations and local ethical requirements in China.

**Reference**

[1] K. Ohtani, Y. Yanagiba, A. Ashimori, A. Takeuchi, N. Takada, M. Togawa, T. Hasegawa, M. Ikeda, N. Miura, Influence of injection timing on severity of cadmium-induced testicular toxicity in mice, The Journal of toxicological sciences, 38 (2013) 145-150.

[2] J. Dai, X. Li, C. Wu, S. Zhang, T. Zhang, D. Zhang, [Effect of different transfection reagents and injection methods in mice testicular injection on the expression of exogenous gene], Sheng wu gong cheng xue bao = Chinese journal of biotechnology, 30 (2014) 1522-1530.

**ADDITIONAL FIGURES**

**FIGURE CAPTIONS**

**Figure S1. Different generation efficiency of PCs in mES cells recovered from cryopreservation with MEF or TM4 feeder**

At day 3, PCs were able to aggregate in group (A) mES+MEF and (B) mES+ TM4. Scale bar = 400 μm.

(B): Number of PCs in group mES+MEF and mES+TM4 was counted under optical microscope (Magnification: 100X). Every sample was counted from 6 independent views (Including the edge of the culture flasks). Results were expressed as mean ± SD. Asterisks indicate statistical significance of differences in the mean of the number of PCs between the two groups (*-P-value <0.05, **-P-value <0.01, ***-P-value <0.001).

**Figure S2. Cell identification by Flow cytometry**

Detection of the cell population by SSC and FSC in group (A) Mature Sertoli cells, and (B) mES+Trans. The gate was set according to the major region of mature Sertoli cells. The results show different cell characteristic between pure mature Sertoli cells and the cells in group mES+Trans.

The cell population was performed using flow cytometry by AMH and FasL antibody. On the top left is AMH^+^/FasL^-^. On the top right is AMH^+^/FasL^+^. On the bottom left is AMH^-^/FasL^-^. On the bottom right is AMH^-^/FasL^+^. The Quad was set according to (C) mature Sertoli cells. (D) and (E) indicate the result of group (No factor) and group (All factors), respectively.

(F): eSCs were identified as AMH+/FasL+ cells. The results show maximum cell portion of generated eSCs among different group, expressed as mean ± SD (n = 3 independent experiments). Asterisks indicate statistical significance of differences in the mean of the number of positive cells between the indicated groups (*-P-value <0.05, **-P-value <0.01, ***-P-value <0.001).

**Figure S3. The FasL-positive cells were sorted from group mES+Trans and identified by AMH and Sox9 antibody in FCM**

AMH and Sox9 are specific markers of eSCs. FCM results indicate these FasL-positive cells sorted from mES+Trans have (A) 91.1% of AMH^-^ positive and (B) 53.3% of Sox9^-^ positive cell population.

(C): The scatter diagram show result of double fluorescence staining method by AMH and Sox9 antibody. The top left is showing AMH^+^/FasL^-^. The top right is showing AMH^+^/FasL^+^. The bottom left is showing AMH^-^/FasL^-^. The bottom right is showing AMH^-^/FasL^+^.

**Figure S4. Isolation of mature Sertoli cells from mice and induced eSCs were injected into mouse testis**

(A) Mature Sertoli cells were isolated from adult mouse testis and (B) FasL^+^ cells (regarded as maximum eSCs) sorted from group mES+Trans were marked with PKH26 showing red fluorescence. Then these labeled cells were respectively injected into mice testis along the testicular efferent ductules. After a week, testis were recovered by surgery. The ST were separated for IF and optical microscopy. Both the mature Sertoli cells and induced eSCs were capable of grow inside the ST without aggregating into colonies. Scale bar = 400 μm.

DDX4 is the marker of SSCs. PGP 9.5 is the marker of sperm. The ST injected with induced eSCs were respectively marked with (C) DDX4 and (D) PGP 9.5 antibody. Positive cells show in dark brown on the section of the ST. The results indicate that the injection of induced eSCs did not obstruct the normal function in testis. Scale bar = 400 μm.

ST = seminiferous tubules


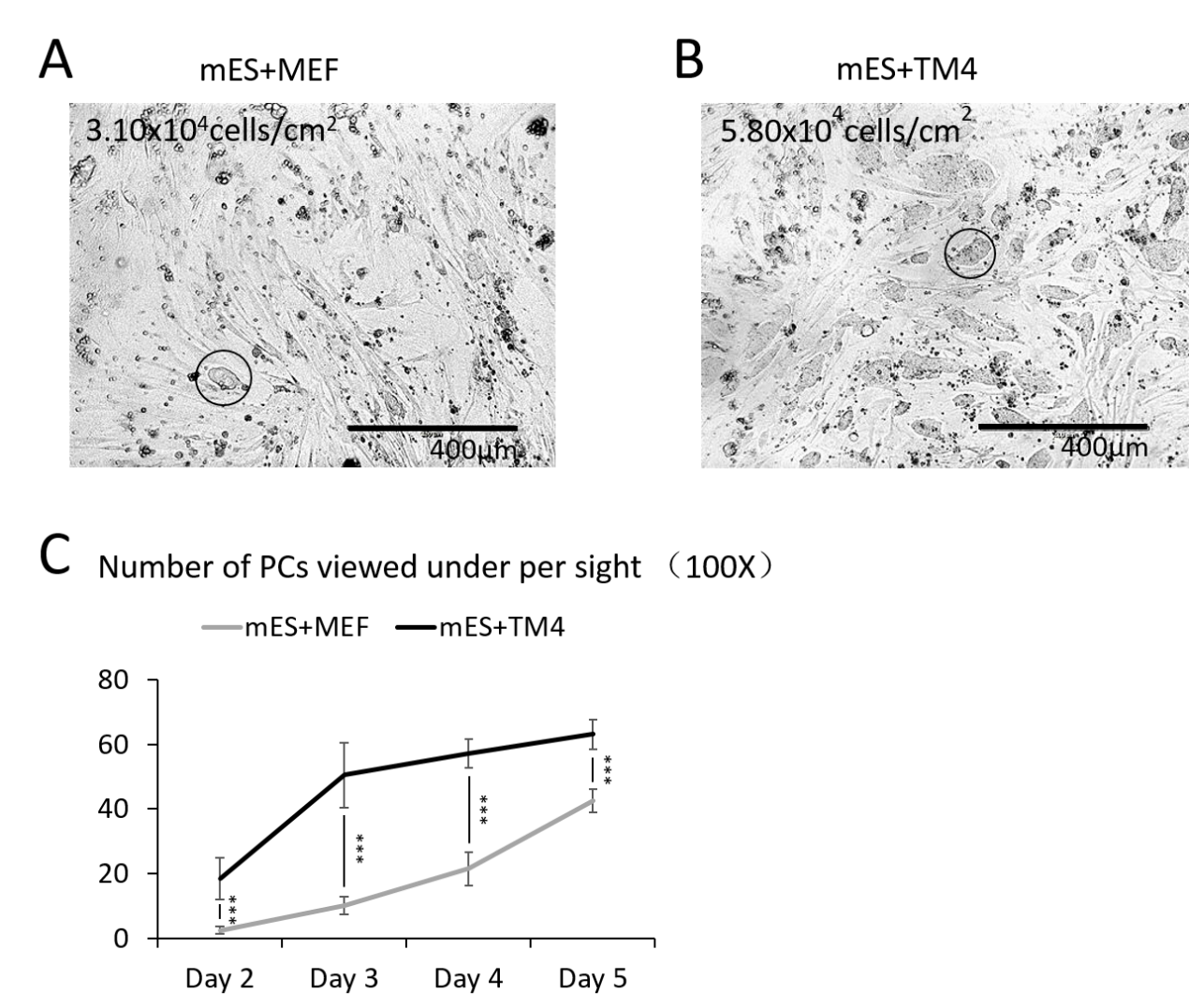


**Figure S1**


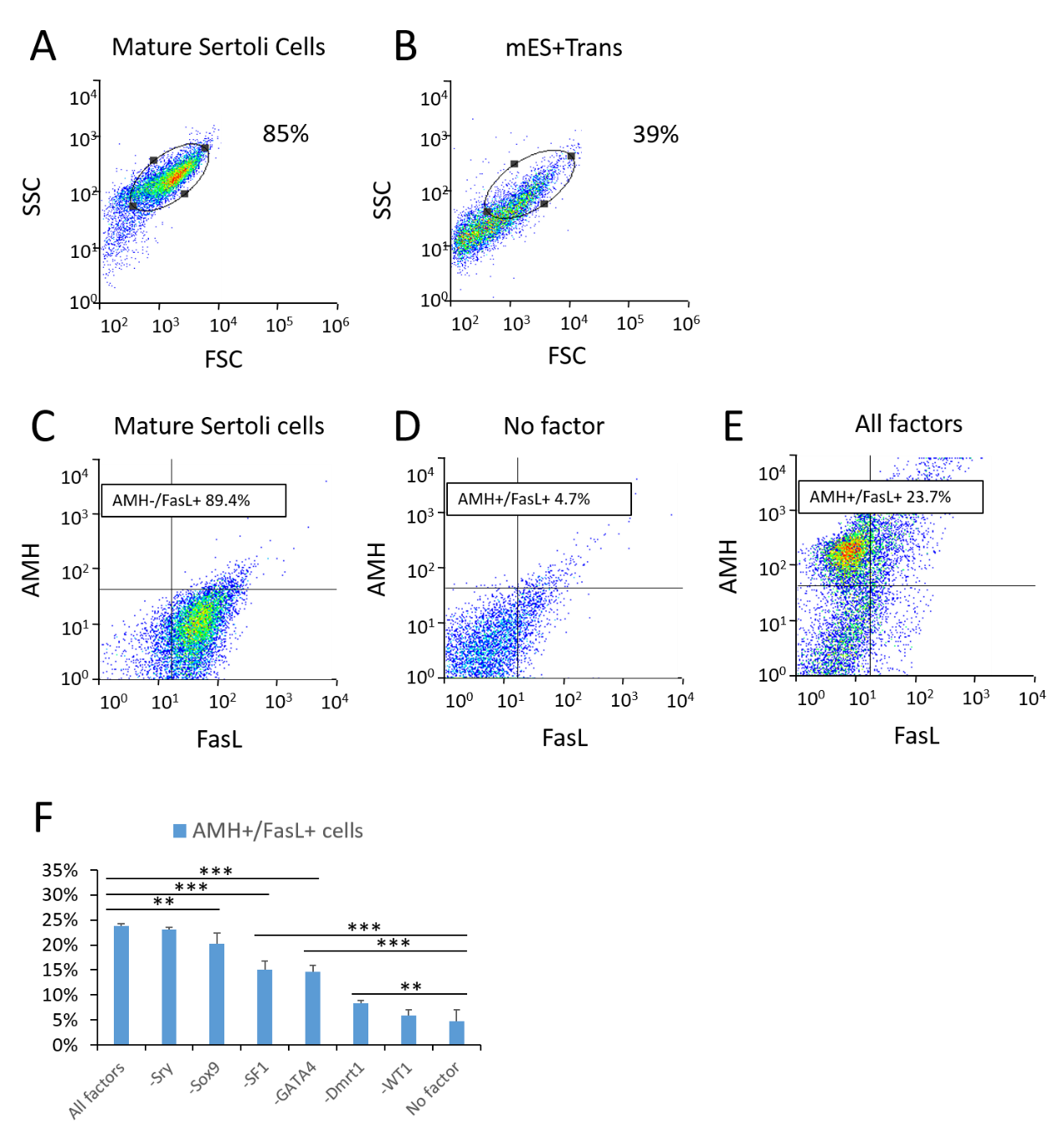


**Figure S2**


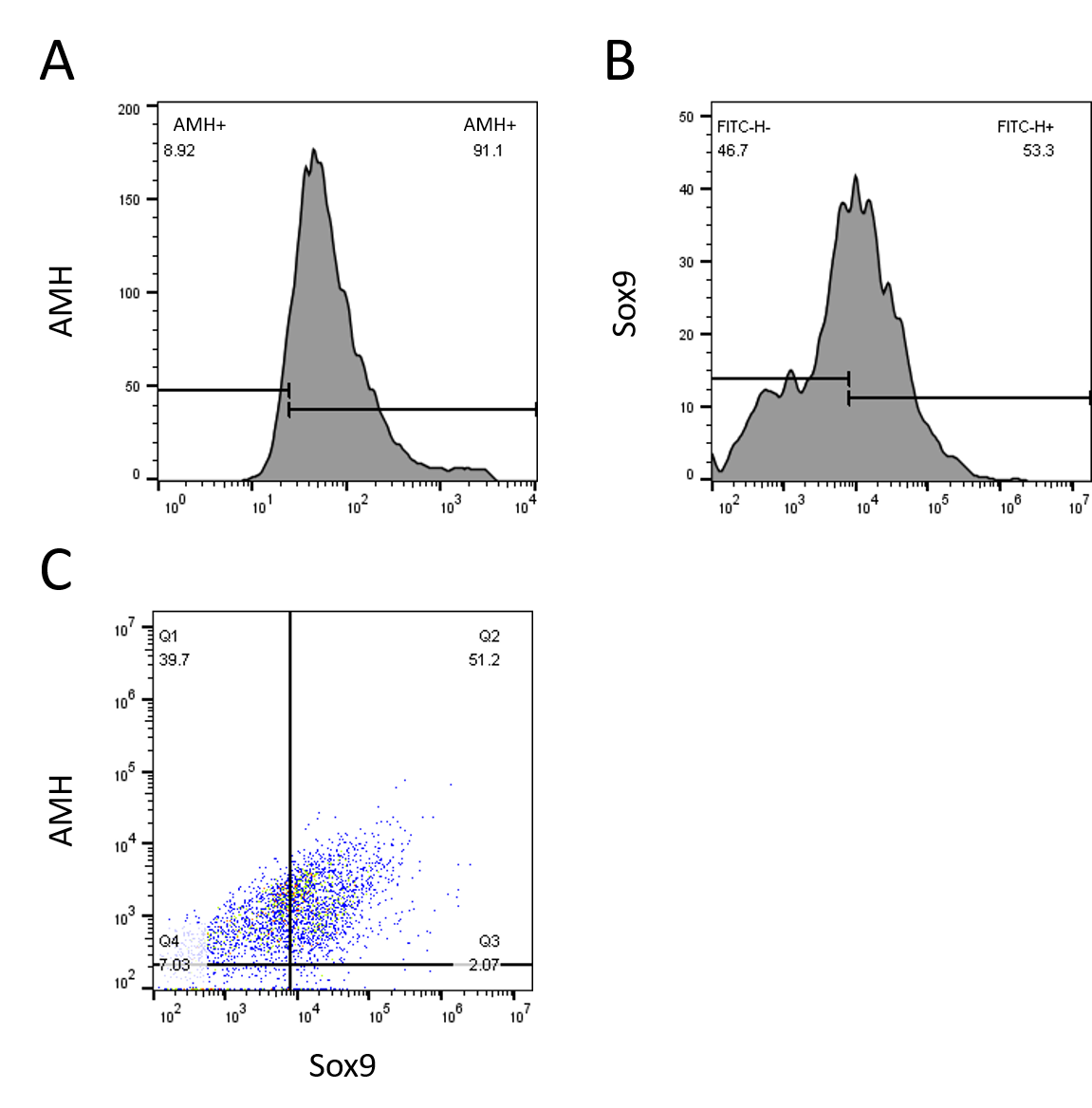


**Figure S3**


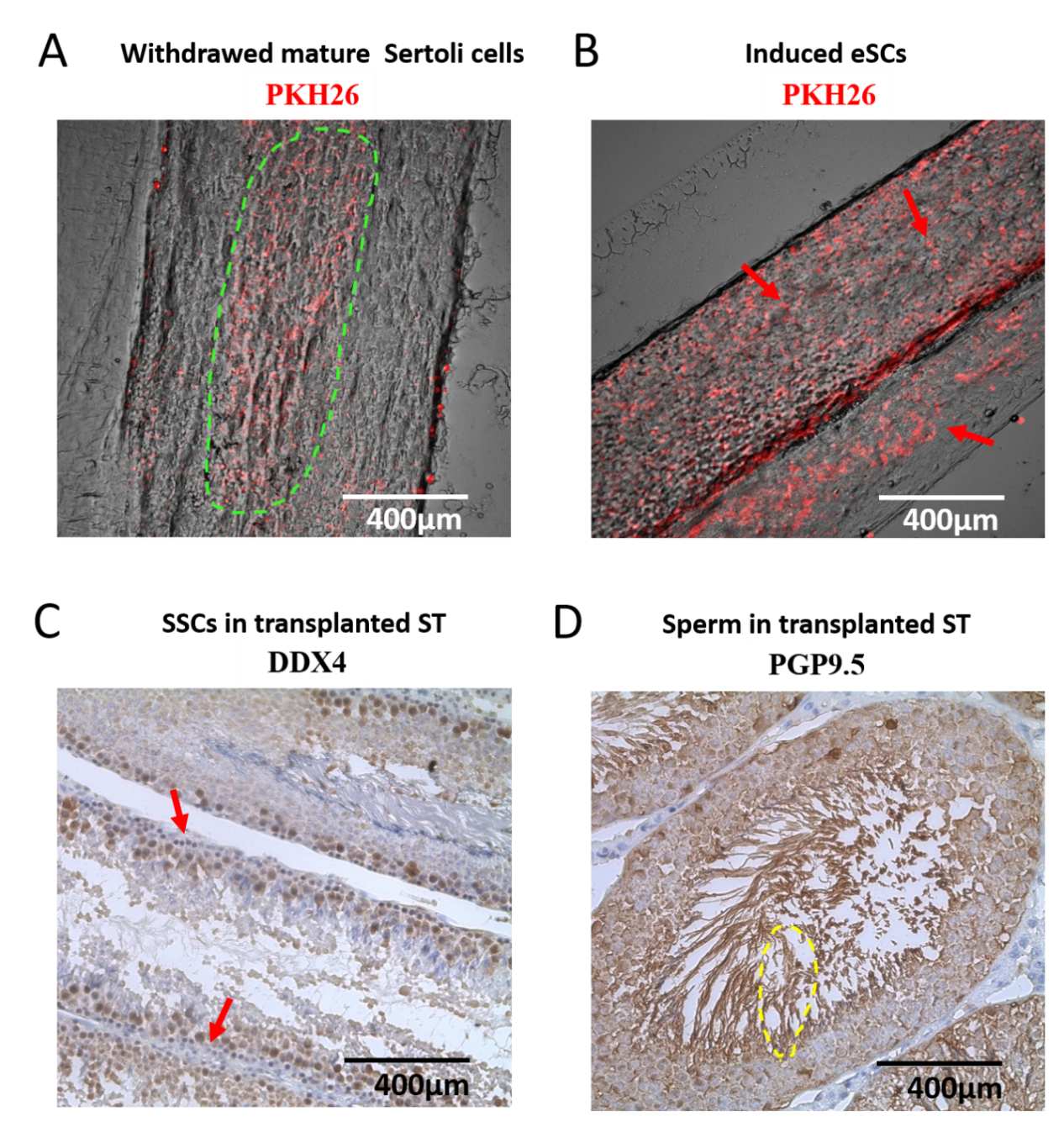


**Figure S4**

**ADDITIONAL TABLES**

**Table S1 Treatment condition of TM4 cells with mitomycin C to suppress proliferation**

| **TM4 cell confluence** | **Concentration of mitomycin C（μg/mL）** | **Duration（hours）** |
| --- | --- | --- |
| 60%--70% | 10 | 2 |
|  | 15 | 2 |
|  | 15 | 3 |
| 70%--80% | 10 | 3 |
|  | 15 | 3 |
|  | 20 | 3 |
| 80%--90% | 15 | 2 |
|  | 15 | 3 |
|  | 20 | 3 |

**Table S2 Primers for complete gene amplification of the target factors**

| **Primer name** | **Forward** | **Reverse** | **Length（bp）** |
| --- | --- | --- | --- |
| Sry | CGGGATCCCGTACCTCCCGGTACAGTTCGC | GGAATTCCTCATGAGACTGCCAACCACA | 1188 |
| Sox9 | CGGGATCCCGACTCGACCTTCAGCCTCTCG | GGAATTCCTGTTTTCACTTTAATGCAAT | 4146 |
| SF1 | CGGGATCCCGTCAGGCGCGTCATTGTTCCT | GGAATTCCGGCTGAGCCATGGTTAAGTC | 2994 |
| WT1 | CGGGATCCCGACACACTTACCTCGCCGGCT | GGAATTCCTTCCTTTTGAATAGACTTTA | 3092 |
| GATA4 | CGGGATCCCGCGCGGCCCTCGTCCCCTGTT | GGAATTCCTCGGAGGAAACAGGAATACA | 3408 |
| Dmrt1 | CGGGATCCCGTCTTTTTTCTTTTTTCTTTT | GGAATTCCGCAGTCATCAATAACGTTTT | 2251 |

**Table S3 Primers for qRT-PCR (quantitative realtime polymerase chain reaction)**

| **Primer name** | **Forward primer** | **Reverse primer** | **Length（bp）** |
| --- | --- | --- | --- |
| Oct4 | GGCTTCAGACTTCGCCTTCT | TGGAAGCTTAGCCAGGTTCG | 99 |
| Sox2 | CAAAAACCGTGATGCCGACT | CGCCCTCAGGTTTTCTCTGT | 92 |
| Nanog | AGCCGTTGGCCTTCAGATAG | AAGTCAGAAGGAAGTGAGCCG | 82 |
| Klf4 | TACCCCTACACTGAGTCCCG | GGAAAGGAGGGTAGTTGGGC | 110 |
| lin28 | CTTTGCCTCCGGACTTCTCT | AAACTGCTGGTTGGACACCG | 101 |
| six1 | CACGCCAGGAGCTCAAACTA | ACCCAAGTCCACCAAACTGG | 132 |
| six4 | CCCCACCGGGCAGATTG | GTCCTTCCGAGGCGCTTT | 71 |
| Lhx9 | GGACCGCTGAATCTAGCACT | CTGCAGCGAGAGATCGAGTT | 141 |
| Emx2 | GGTTTCAGAACCGGAGAACG | CTATTTCCTCCGGACTCGCC | 138 |
| GATA4 | AGCAGGACTCTTGGAACAGC | GCCCCAGCCTTTTACTTTGC | 138 |
| WT1 | ATCCCAGGCAGGAAAGTGTG | GTGCTGTCTTGGAAGTCGGA | 111 |
| SF1 | GCCCGAGTGGCCGTC | TGTCACCACACACTGGACAC | 148 |
| Sry | TGGTGAGAGGCACAAGTTGG | AGGCTTTTCCACCTGCATCC | 87 |
| Sox9 | GTGCAAGCTGGCAAAGTTGA | TGCTCAGTTCACCGATGTCC | 106 |
| Dmrt1 | GGATCCCCCGTGAAGAACAG | GAGGCCCGTAGTATGAGTGC | 142 |
| lin28 | CTTTGCCTCCGGACTTCTCT | AAACTGCTGGTTGGACACCG | 101 |
| KitL | CAGCGCTGCCTTTCCTTATG | TCCCGGAGCGATTTTCTTGG | 89 |
| Klf4 | TACCCCTACACTGAGTCCCG | GGAAAGGAGGGTAGTTGGGC | 110 |
| AMH | CTACTCAAGGACAGCTCAGGC | GGAGGCGACTGTCTCAAAGG | 78 |
| SF1 | GCCCGAGTGGCCGTC | TGTCACCACACACTGGACAC | 148 |
| FOG2 | CCTAACTGAAGAAGCCGCCA | AGGCGCACATATAGCAGTCC | 105 |
| Sry | TGGTGAGAGGCACAAGTTGG | AGGCTTTTCCACCTGCATCC | 87 |
| Sox8 | GATACATCCAACCCGGGGAC | GCTGGGAAGAACGGTGGTAA | 76 |
| Dhh | TGGCTTGGGTGTATCTGTGG | AACGAGACCGGGAAAGAACG | 111 |
| ISL1 | ATCGAGTGTTTCCGCTGTGT | AAGGGACTGAGAGGGTCTCC | 146 |
| Sox2 | CAAAAACCGTGATGCCGACT | CGCCCTCAGGTTTTCTCTGT | 92 |
| Nanog | AGCCGTTGGCCTTCAGATAG | AAGTCAGAAGGAAGTGAGCCG | 82 |
| Klf4 | TACCCCTACACTGAGTCCCG | GGAAAGGAGGGTAGTTGGGC | 110 |
| lin28 | CTTTGCCTCCGGACTTCTCT | AAACTGCTGGTTGGACACCG | 101 |
| Emx2 | GGTTTCAGAACCGGAGAACG | CTATTTCCTCCGGACTCGCC | 138 |
| FOG2 | CCTAACTGAAGAAGCCGCCA | AGGCGCACATATAGCAGTCC | 105 |
| AMH | CTACTCAAGGACAGCTCAGGC | GGAGGCGACTGTCTCAAAGG | 78 |
| kitL | CAGCGCTGCCTTTCCTTATG | TCCCGGAGCGATTTTCTTGG | 89 |
| Dhh | TGGCTTGGGTGTATCTGTGG | AACGAGACCGGGAAAGAACG | 111 |
| Ptgds | GGCTCCTTCTGCCCAGTTTTC | CCAGGAGGACCAAACCCATC | 120 |
| GDNF | TGTTCCGCGCTTCTTCTTCT | TCCTTCCTCCTCCGAGTGTC | 120 |
| Fgf9 | ACGGTCGGATGGGATGAAGA | TGGCACAGGTTCAAGGTCAA | 147 |
| Cbx2 | GGAGTACCTGGTCAAGTGGC | GCCTCGGGTCCAAAATGTTC | 80 |
| Col9a1 | AAGAAGGAGCAAGCTTGGGG | CCAGTGGGTTCTCTGTGGTT | 125 |
| Sox8 | GATACATCCAACCCGGGGAC | GCTGGGAAGAACGGTGGTAA | 76 |

**Table S4 Lentivirus producing and packaging system**

| **Size** | **FUW-TetO + target gene（μg）** | **PAX2**  **（μg）** | **PMD.2G**  **（μg）** |
| --- | --- | --- | --- |
| 24 well plate | 0.25 | 0.187 | 0.062 |
| 12 well plate | 0.5 | 0.375 | 0.125 |
| 6 well plate | 1 | 0.75 | 0.25 |
| T25 flask | 2 | 1.5 | 0.5 |
| T75 flask | 6 | 4.5 | 1.5 |

**Table S5 Antibodies for IF and ICC**

| **Antibodies** | **Description** | **Producer** |
| --- | --- | --- |
| FasLantibody（Kay-10）: sc-19988 | mouse monoclonal IgG_2b_ (kappa light chain)  Application: IF, ICC and FCM | Santa Cruz |
| Anti-AMH antibody C -terminal: ab229212 | Rabbit polyclonal  Application: IF, ICC | Abcam |
| m-IgGk BP-HRP | mouse IgGk binding protein-HRP  Application: ICC | Santa Cruz |
| m-IgGk BP-FITC: sc-516140 | mouse IgG kappa binding protein conjugated to fluorescein (FITC)  Application: IF, FCM | Santa Cruz |
| mouse anti-rabbit IgG-CFL 488: sc-516248 | mouse monoclonal secondary antibody conjugated to CruzFluor^TM^ 488  Application: IF, ICC, FCM | Santa Cruz |

IF (Immunofluorescence); IHC (Immunohistochemistry); FCM (Flow cytometry)

**Table S6 Antibodies for development stage identification by FCM**

| **Antibodies** | **Description** | **Producer** |
| --- | --- | --- |
| AMH antibody: orb40644 | Primary antibody: Rabbit polyclonal unconjugated | Biorbyt |
| CD178 (Fas Ligand) | Primary antibody: Armenian hamster monoclonal antibody (MFL3) conjugated to APC | eBioscience |
| Nr5a1 antibody (PA5-25030) | Primary antibody: Rabbit polyclonal unconjugated | Invitrogen |
| Anti-Emx2 antibody (ab94713) | Primary antibody: Rabbit polyclonal unconjugated | Abcam |
| Anti-Sox9 antibody (ab185966) | Primary antibody: Rabbit monoclonal [EPR14335-78] | Abcam |
| Mouse anti-rabbit IgG-FITC: sc-2359 | Second antiboy: Mouse monoclonal conjugated to FITC | Santa Cruz |

FCM (Flow cytometry)
